# Radioisotope-Guided Excision of Mediastinal Lymph Nodes in Patients with Non-Small Cell Lung Carcinoma: Feasibility and Clinical Impact

**DOI:** 10.3390/cancers15133320

**Published:** 2023-06-24

**Authors:** Cristiano Pini, Edoardo Bottoni, Francesco Fiz, Veronica Maria Giudici, Marco Alloisio, Alberto Testori, Marcello Rodari, Martina Sollini, Arturo Chiti, Umberto Cariboni, Lidija Antunovic

**Affiliations:** 1 Department of Biomedical Sciences, Humanitas University, Via Rita Levi Montalcini 4, Pieve Emanuele, 20072 Milan, Italy; cristiano.pini@cancercenter.humanitas.it (C.P.); veronica.giudici@cancercenter.humanitas.it (V.M.G.); chiti.arturo@hsr.it (A.C.); 2 Diagnostic Imaging Department, IRCCS Humanitas Research Hospital, Rozzano, 20089 Milan, Italy; marcello.rodari@cancercenter.humanitas.it (M.R.); antunovic.lidija@hsr.it (L.A.); 3 Division of Thoracic Surgery, IRCCS Humanitas Research Hospital, Via Manzoni 56, 20089 Rozzano, Italy; edoardo.bottoni@humanitas.it (E.B.); alberto.testori@cancercenter.humanitas.it (A.T.); umberto.cariboni@cancercenter.humanitas.it (U.C.); 4 Nuclear Medicine Unit, Department of Diagnostic Imaging, Ente Ospedaliero “Ospedali Galliera”, 16128 Genoa, Italy; 5 Department of Nuclear Medicine and Clinical Molecular Imaging, University Hospital Tübingen, 72076 Tübingen, Germany

**Keywords:** lung carcinoma, mediastinal lymphadenectomy, sentinel lymph node, radio-guided surgery, nuclear medicine

## Abstract

**Simple Summary:**

We tried to use a small amount of radioactivity, injected around non-small cell lung cancers, to see if this technique can identify the lymph node receiving the flow (and thus potentially the metastases) from the tumour. Our results show that it is possible to do so; this technique could be used to obtain a personalised and potentially safer approach in lung cancer surgery.

**Abstract:**

Background: Intraoperative localisation of nodal disease in non-small cell lung cancer (NSCLC) can be challenging. Lymph node localisation via radiopharmaceuticals is used in many conditions; we tested the feasibility of this approach in NSCLC. Methods: NSCLC patients were prospectively recruited. Intraoperative peri-tumoral injections of [99mTc]Tc-albumin nanocolloids were performed, followed by removing the tumour and locoregional lymph nodes. These were examined ex vivo with a gamma probe and labelled sentinel lymph nodes (SLNs) if they showed any activity or non-sentinel lymph nodes (nSLNs) if they did not. Thereafter, the surgical field was scanned with the probe; any further radioactive lymph node was removed and labelled as “extra” SLNs (eSLNs). All specimens were sent to histology, and metastatic status was recorded. Results: 48 patients were enrolled, and 290 nodal stations were identified: 179 SLNs, 87 nSLNs, and 24 eSLNs. A total of 44 nodal metastases were identified in 22 patients, with 36 of them (82%) located within SLNs. Patients with nSLNs metastases had at least a co-existing positive SLN. No metastases were found in eSLNs. Conclusions: The technique shows high sensitivity for intraoperative nodal metastases identification. This information could allow selective lymphadenectomies in low-risk patients or more aggressive approaches in high-risk patients.

## 1. Introduction

Non-small cell lung carcinoma (NSCLC) is among the most common and lethal neoplasms, representing the leading cause of cancer-related death in Western countries [1]. NSCLC is characterised by an early asymptomatic phase, which hinders disease identification in its earliest stages [2]. Consequently, NSCLC is often diagnosed at a clinical stage, when the potentially curative surgical approach is challenging [3]. The main tools for the NSCLC workup are contrast-enhanced CT, optimal in defining the extension of the primary tumour, and [18F]Fluorodeoxygluocose ([18F]FDG) PET/CT, characterised by a great sensitivity in detecting nodal metastases and in ruling out remote localisations [4,5,6]. In general, NSCLCs up to stage IIIA are routinely considered for surgical treatment; in particular, nodal localisations in the homolateral hilus (N1) and underneath the carina (N2) are considered surgically manageable [7]. However, higher stages can also be considered for surgery, in high-volume centres and after a thorough multidisciplinary case-by-case evaluation [8,9,10]. Regrettably, despite aggressive approaches, the prognosis of NSCLC patients with nodal disease remains dismal [11].

The effectiveness of the surgical approach relies on the accurate identification of all disease foci and the subsequent attainability of a radical result. However, pre-operative staging based on medical imaging does not guarantee perfect sensitivity; in particular, [18F]FDG PET/CT is limited by the intrinsic spatial resolution of the technique and cannot detect micro-metastases reliably [12]. Moreover, smaller lymph nodes might be challenging to identify during the intervention [13]. Due to these limitations, current standards of care guidelines prudentially recommend extensive mediastinal lymph node dissections even in early-stage cancers; this procedure can be technically demanding and may increase the rate of surgical complications and prolong hospitalisation.

It should be borne in mind that the process of nodal spread is not of random nature. Tumour cells are collected by the local lymphatic vessels, and then transported to the closest mediastinal nodes. This process can be tracked using specific radiopharmaceuticals, such as radioisotope-labelled colloids which, following peritumoral injection, quickly travel and become trapped within the local lymph nodes. By employing a radioisotope probe during surgery, these first-line nodes (also called sentinel lymph nodes, SLNs) can be identified [14].

The intraoperative identification of SLNs via radiopharmaceuticals has been employed in several tumour types successfully, most commonly in breast cancer and melanoma, where this technique revolutionised the standard-of-care and surgical management [15,16]. However, this technique has yet to gain traction in the NSCLC setting due to the higher complexity of the mediastinal node network, and the technical challenge associated with intra-operative radiopharmaceutical injection. Moreover, most attempts so far only included early-stage forms, so our knowledge of the sentinel node pathing in the advanced disease is limited. In this context, we set up a prospective, single-centre study in which we tested the radiopharmaceutical-guided SLN approach in patients eligible for surgical treatment of NSCLC of all surgically amenable stages, with the aim to evaluate the feasibility and accuracy of this technique.

## 2. Materials and Methods

### 2.1. Study Design and Population

Patients referring to IRCCS Humanitas Research Hospital for surgery in NSCLC were prospectively and consecutively recruited between May 2021 and April 2022. The enrolment requirements included a histologically confirmed diagnosis of NSCLC and the completion of the pre-operatory staging with CT and [18F]FDG PET/CT. All patients signed a study-specific informed consent module, and their enrolment was subject to approval from the local ethical committee (approval number 336/21, 20 April 2021). For all patients, data relative to demographics, clinical history, and tumour stage according to the eighth version of the American Joint Committee on Cancer TNM system and pathology, were collected from the electronic clinical medical records.

### 2.2. Lymphoscintigraphy and Surgery

During surgery, immediately after the identification of the primary tumour site, four intraparenchymal peri-tumoral injections of [99mTc]Tc-Albumin Nanocolloids (Nanotop^®^, ROTOP Pharmaka AG, Dresden, Germany) were performed. Starting from 10 min after the injection, the surgical removal of the primary tumour and locoregional lymph nodes was performed according to the best clinical practices.

**Figure 1:**
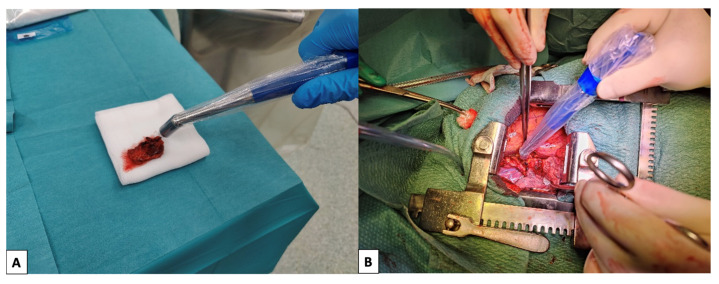
Counting of lymph nodal activity ex vivo (**A**) and control of the surgical field with gamma-probe (**B**).

All the excised lymph nodes were examined with a gamma probe (NeoProbe^®^ Gamma Detection System, Devicor Medical Products, Inc., Cincinnati, OH, USA or Gamma Finder II^®^, World of Medicine GmbH, Berlin, Germany) ex vivo (Figure 1A) and considered as sentinel lymph nodes (SLNs) if they showed a significant activity; in particular, the activity was deemed significant when the uptake count of the specimen was higher than the background. The remaining ones, showing no significant activity, were considered non-sentinel lymph nodes (nSLN). Thereafter, at the end of the standard-of-care surgical excision of the primary tumour and locoregional lymph nodes, the surgical field was scanned with the gamma probe (Figure 1B), and any further radioactive lymph node was removed; these were labelled as “extra” SLNs (eSLNs). All uptake counts, and timings of the measurements, were recorded. All nSLNs, SLNs and eSLNs were sent to definitive histology, and the presence or absence of metastases was documented.

### 2.3. Statistical Analysis

Data are presented as mean and interquartile range (IQR), unless otherwise specified. Predictors of metastases in SLNs, nSLNs, and eSLNs were analysed via a univariate binary logistic regression model; further multinomial logistic regression analysis was conducted on selected factors based on significance level at the univariate testing and clinical reasoning. SPSS v. 24 (IBM, Armonk, NY, USA) was used for the analyses. A *p*-value of <0.05 was considered significant.

## 3. Results

### 3.1. Patients’ Population

A total of 48 patients were prospectively enrolled. In higher prevalence, they were males (65%), with smoking habits (77%), and a histologically confirmed diagnosis of lung adenocarcinoma (71%). Baseline patients’ characteristics and histopathological staging details are described in Table 1.

### 3.2. Distribution of Metastases in SLNs and nSLNs

During surgery, a total of 290 lymph-nodal stations were dissected, including 179 SLNs, 87 nSLNs, and 24 eSLNs. A total of 44 metastatic lymph-nodal stations were identified in 22 patients. A total of 36 out of these affected stations (82%) were located within SLNs. In three patients, however, there was a co-existence of metastases within and without SLNs. Particularly, one of these patients had a single nSLN metastasis, one had a mixture of SLNs and nSLNs metastases, while the last one had a single positive SLN and evidence of diffuse neoplastic lymphangitis, which probably hindered the tracer diffusion to the proper sentinel localisations (Table 2). Five, nine, and thirty metastases were identified in patients with cN0/cN1/cN2 status, respectively. In two cases, nodal metastases were identified in a mediastinal localisation with no evidence of hilar/intrapulmonary disease; in both cases, the nodal localisations were within SLNs. See Table 3 for details. Finally, there was no significant correlation between count intensity and the presence or absence of metastasis within SLNs.

### 3.3. Lymphatic Pathing

In typical upper lobe resections (26 procedures), only in 2 cases were metastases found in sub-carinal lymph nodes, and in both cases they were SLNs; in 6 cases, sub-carinal SLNs were pathologically negative; in 18 cases, pathologically negative sub-carinal nodes were nSLNs. In typical lower lobe resections (10 procedures), no metastases were located within higher para-tracheal lymph nodes; in 5 cases, pathologically negative high para-tracheal nodes were located within SLNs areas, and in the remaining 5 cases in nSLNs.

### 3.4. Predictors of Lymph Nodes Localisations and of eSLNs

Predictors of the presence of positive sentinel lymph nodes are depicted in Supplemental Table S1 (univariate analysis) and Table 4 (multivariate model): male gender and history of neoadjuvant chemotherapy showed a trend for increasing the risk, yet the presence of clinically positive lymph node in N2 localisations were the only definite predictor of this occurrence.

Given the rarity of its occurrence, no single parameter was correlated with presence of metastases within non-sentinel lymph nodes, even if cN positivity showed some degree of association (Supplemental Table S2). Therefore, no multivariate analysis was attempted.

Finally, a longer uptake time (greater than the median value) was the sole predictor inversely correlated with the presence of eSLNs on both univariate (Supplemental Table S3) and multivariate analysis (Table 5).

## 4. Discussion

The present study allowed for the gaining of a number of insights into the potential role of the sentinel lymph node technique in non-small-cell lung cancer. This technique resulted in safe, easily implementable, and accurate identification of nodal disease. In fact, disease localisations were found within sentinel nodes or, in rare instances, in non-sentinel lymph node stations located farther down the line of positive lymph nodes. These data support the use of the technique to identify nodal localisations reliably, or to prompt further exploration in case of positive sentinel localisations. Some guidelines advocate for lobe-specific mediastinal lymph node dissection, with the rationale being that there is an obvious correlation between the site of lymph-nodal metastasis and the location of the primary tumour [17]. In particular, in upper-lobe tumours, there is a significantly higher incidence of metastasis within superior mediastinal lymph nodes; conversely, lower-lobe tumours tend to spread to the inferior and sub-carinal nodal stations. This reasoning could justify a targeted approach to lymph-nodal dissection omitting the complete sub-carinal nodal resection in the surgical treatment of upper-lobe NSCLC [18]. Nonetheless, in some records, nearly 6% of patients treated according to the lobe-specific dissection might have had their metastatic lymph nodes missed by sampling stations not included in the lobe-specific protocol [17]. In any case, most guidelines agree that sub-carinal lymph nodes should at least always be sampled, and the European Society of Thoracic Surgeons (ESTS) guidelines have defined systematic nodal dissection as requiring the dissection and removal of all mediastinal tissue containing lymph nodes within precise anatomical landmarks [19]. Our analysis further highlighted how the clinical N+ status on the staging exams affects the likelihood of nodal metastasization. Combining the information from the pre-surgical staging imaging with that of sentinel node biopsy might help in guiding the surgical decision-making as to whether or not to sample the more remote lymph nodes. Patients treated with neo-adjuvant therapy might also be more likely to bear nodal localisations, given their more advanced disease.

In our series, retrieving “extra” sentinel nodes, i.e., those that the surgeon would not have removed if they had not had the sentinel node technique available, did not change the staging or improve the radicality of the intervention. However, such sentinel nodes were mostly present in patients with no evidence of nodal metastases. It might be interesting to further test the concept of additional lymph node identification in higher stages NSCLC, with a higher likelihood of node spread. Moreover, the current study was carried out in a high-volume referral centre; it might be speculated that using the sentinel node method to detect more lymph nodes than those identified by the surgeon might benefit operators still in the earlier phases of the learning curve. On the other hand, it must also be considered that the main factor inversely correlating to the presence of “extra” lymph nodes was the time elapsed between the tracer injection and the conclusion of the regular surgical procedure. In this sense, it could be hypothesised that a longer surgical procedure might allow the operators to perform a more complete lymphadenectomy, comprising all nodal stations reached by the radiopharmaceutical.

Our results about the intraoperative detection rate are in line with those presented in the literature, even if the large majority of them analysed early-stage NSCLC [20,21,22]; nonetheless, some smaller reports included higher-stage tumours as well [23]. The novelty of our work also resides in the fact that we analysed a wide range of NSCLC staging, including those with higher stages. We found that, especially in patients with clinically evident metastatic spread, the SLN technique can be of value in identifying patients with diffuse nodal disease, or in which the lymphatic flow does not follow the expected pattern. Our data suggest that more advanced and aggressive tumours might present unique challenges; in particular, massive metastastization or neoplastic lymphangitis might prevent an accurate nodal mapping with the pharmaceutical. In these cases, the method might underestimate the disease extent, and more aggressive surgical excision should be carried out.

In general, however, the information gathered thanks to this method could inform more selective lymphadenectomies, especially in early-stage lung cancers, reducing the surgical burden for operators and patients and driving a paradigm shift towards less invasive approaches [24]. Conversely, the added sensitivity provided by the intraoperative mapping of the locoregional lymphatic system can improve the identification of hard-to-spot nodes, especially in more advanced diseases, empowering more radical interventions, reducing the rate of relapses and improving survival. Finally, it has been suggested that employing the sentinel lymph node techniques might reduce the surgery time and curb its overall costs [25].

These results might prompt further targeted investigations. On the one hand, higher-stage tumours, which are those more prone to relapse and progression, might benefit from the sentinel lymph node approach the most. Studying the behaviour of the nodal spread in these patients might improve our capability to offer a radical treatment, even in these aggressive forms. On the other hand, prospective investigations assessing the possibility of more limited nodal resection in early-stage NSCLC might help reduce the rate of complications and postoperative morbidity [26,27]. Finally, even higher detection rates could be reached by switching from the one-dimensional exploration capability afforded by the gamma-probe to the 2D imaging offered by portable gamma cameras [28] or even to 3D, in vivo representation of sentinel nodes following pre-operative SPECT imaging, which has been suggested in many surgical fields [29,30].

From the feasibility point of view, we found that this technique can be implemented easily, with a very fast learning curve for the surgical team. An important advantage of this method is that it does not entail novel or hard-to-setup radiopharmaceutical: the lymphatic mapping can be prepared in minutes with minimal notice beforehand. Moreover, the carrier molecule is cheap and widely available; the dose absorbed by the patients as well as the surgical team is negligible.

Our study is not without limitations. The studied population is relatively small, even if the sample size is in line with or larger than the existing literature. We recruited patients with all NSCLC stages amenable to surgery. Thus, our results might not always be representative of every single stage. Nonetheless, this study was conceived to shed light on the lymphatic pathing of all lung cancer stages; the consistency of our results with those of the existing literature suggests that the sentinel node mapping can be applied safely to higher stages as well. The role of this method in patients with a clinical N2 status could be limited, since these patients should undergo a full excision in all cases; however, performing a sentinel node mapping could be helpful in those cases were the cN2 staging is conflicting across methods or unclear.

Finally, follow-up data of the studied patients are not available yet; the correlation between the sentinel node status and the long-term outcome should be assessed as soon as this information becomes available.

## 5. Conclusions

Sentinel node mapping with radiolabelled colloids in NSCLC is a safe procedure in all stages, able to identify nodal metastases in most cases. The identification of metastasization in a sentinel lymph node should, however, prompt a radical excision of all the nearby nodal stations, especially when an obstacle in the lymphatic drainage is suspected. On the other hand, identifying sentinel lymph nodes outside of the standard operating field does not seem to bring advantages in metastases identification.

## Figures and Tables

**Table 1:** Characteristics of the patients included in the study.

Patients, n	48		Mean injected activity, MBq (IQR)	31.5 (5)	
Sex, n	17 F (35%)	31 M (65%)			
Mean age, years (IQR)	69 (12)		Dissected lymph nodes, n	Total	290
				SLNs	179 (62%)
Smoking habit, n	Yes	37 (77%)		nSLNs	87 (30%)
	No	11 (23%)		eSLNs	24 (8%)
Histoype, n	Adenocarcinoma	34 (71%)	Metastatic lymph nodes, n	Total	44
	Squamous cells	14 (29%)		SLNs	36 (82%)
Neoadjuvant treatment, n	Yes	10 (21%)		nSLNs	8 (18%)
	No	38 (79%)		eSLNs	0 (0%)
Clinical T (cT) staging, n	cT1	14 (29%)	Pathological T (pT) staging, n	pT1	15 (31%)
	cT2	16 (33%)		pT2	18 (38%)
	cT3	15 (31%)		pT3	9 (19%)
	cT4	3 (6%)		pT4	6 (12%)
Clinical N (cN) staging, n	cN0	25 (52%)	Pathological N (pN) staging, n	pN0	26 (54%)
	cN1	10 (21%)		pN1	9 (19%)
	cN2	13 (27%)		pN2	13 (27%)

IQR: interquartile range.

**Table 2:** Uptake patterns in patients with metastases in both SLNs and nSLNs.

Patient #	Primary Localization	Histotype	cTNM, yTNM	Positive SLN Stations	Positive nSLN Stations	Negative SLN Stations
31	Left lung	Squamous cells	cT2N2	Within the left lung	Subaortic	Left hilus, subcarinal, paraesophageal
37	Left upper lobe	Adenocarcinoma	cT2N1	Subaortic	Para-aortic, left hilus, subcarinal, paraesophageal	Within the left upper lobe
47	Right upper lobe	Adenocarcinoma	cT3N2, yT2N2	Within the right upper lobe, upper/lower tracheal right, subcarinal	Anterior mediastinum, upper tracheal right, upper tracheal corner	Lower tracheal left

**#** consecutive patient number.

**Table 3:** Distribution of nodal metastases according to clinical staging.

Nodal Staging	Number of Patients	Number of Metastases in SLNs	Number of Metastases in nSLNs	Skip Metastases
cN0	25	5	None	1 *
cN1	10	5	4	None
cN2	13	26	4	1 *

* found in an SLN.

**Table 4:** Multivariate analysis of positive SLN predictors.

Parameter	OR	Significance	LB	UB
Sex (male)	8.273	0.065	0.875	78.204
Neoadjuvant chemotherapy	12.882	0.078	0.753	220.279
cN0	Reference			
cN1	9.665	0.083	0.746	125.195
cN2	17.506	0.021	1.548	198.031

OR: odds ratio; LB: lower bound; UB: upper bound.

**Table 5:** Multivariate analysis of predictors of “extra” sentinel lymph nodes.

Parameter	OR	Significance	LB	UB
Neoadjuvant chemotherapy	0.17	0.159	0.015	1.996
Uptake time (>median)	0.227	0.029	0.06	0.862
cN0	Reference			
cN1	0.737	0.783	0.084	6.439
cN2	0.653	0.645	0.107	4.1

OR: odds ratio; LB: lower bound; UB: upper bound.

## Data Availability

The datasets generated during and/or analysed during the current study are available from the corresponding author upon reasonable request.

## Supplementary Materials

The following supporting information can be downloaded at: https://www.mdpi.com/article/10.3390/cancers15133320/s1, Supplemental Table S1: Univariate predictors of SLN metastases; Table S2: Univariate predictors of nSLN metastases; Table S3. Univariate predictors of eSLN presence.
