# Supplementary material for: Radioisotope-Guided Excision of Mediastinal Lymph Nodes in Patients with Non-Small Cell Lung Carcinoma: Feasibility and Clinical Impact
Source: Cancers (Basel). 2023 Jun 24;15(13):3320. doi: 10.3390/cancers15133320 (PMC10341198; doi:10.3390/cancers15133320)
Supplement: Supplementary file 1 [file cancers-15-03320-s001.zip › cancers-2436301-supplementary.pdf]

Supplemental Material

Table S1. Univariate predictors of SLN metastases.

Parameter	OR	Significance	LB	UB
Age (>median)	1	1	0,321	3,113
Sex (male)	2,914	0,096	0,826	10,279
Smoking	1,658	0,475	0,414	6,639
Comorbidity	1,185	0,791	0,338	4,156
Histology (adenocarcinoma)	0,525	0,316	0,149	1,85
Neoadjuvant treatment	17,308	0,01	1,972	151,884
cT	1,645	0,135	0,857	3,157
cN	4,864	<0,001	2	11,831
Injected activity (>median)	1,333	0,625	0,421	4,222
Uptake time (>median)	1,273	0,681	0,403	4,019
pT	0,938	0,827	0,528	1,665
pN	205,213	<0,001	11,939	3527,227
Tumour size (>median)	1	1	0,321	3,113

Table S2. Univariate predictors of nSLN metastases.

Parameter	OR	Significance	LB	UB
Age (>median)	-	n/d	-	-
Sex (male)	1,103	0,938	0,093	13,135
Smoking	0,571	0,661	0,047	6,970
Comorbidity	-	n/d	-	-
Histology (adenocarcinoma)	0,813	0,87	0,068	9,756
Neoadjuvant treatment	2	0,588	0,163	24,588
cT	1,268	0,714	0,365	4,522
cN	4,398	0,114	0,702	27,551
Injected activity (>median)	-	n/d	-	-
Uptake time (>median)	2,4	0,488	0,202	28,451
pT	0,869	0,822	0,256	2,948
pN	4,358	0,11	0,716	26,511
Tumour size (>median)	>1000	0,998	-0,1	>1000

Table S3. Univariate predictors of eSLN presence.

Parameter	OR	Significance	LB	UB
Age (>median)	2	0,244	0,623	6,421
Sex (male)	0,489	0,224	0,147	1,629
Smoking	0,818	0,772	0,211	3,176
Comorbidity	1,421	0,592	0,393	5,141
Histology (adenocarcinoma)	0,933	0,915	0,265	3,289
Neoadjuvant treatment	0,278	0,134	0,052	1,483
cT	0,737	0,352	0,387	1,403
cN	0,613	0,175	0,302	1,245

Injected activity (>median)	1,264	0,692	0,395	4,043
Uptake time (>median)	0,231	0,024	0,065	0,824
pT	0,801	0,463	0,445	1,448
pN	0,65	0,227	0,323	1,307
Tumour size (>median)	0,709	0,559	0,224	2,245
